# Bond Strength, Microleakage, Microgaps, and Marginal Adaptation of Self-adhesive Resin Composites to Tooth Substrates with and without Preconditioning with Universal Adhesives

**DOI:** 10.3290/j.jad.b4949691

**Published:** 2024-02-08

**Authors:** Alaaeldin Elraggal, Islam Abdel Raheem, Ahmed Holiel, Abdulaziz Alhotan, Abdulrahman Alshabib, Nikolaos Silikas, David C. Watts, Nada Alharbi, Rania R. Afifi

**Affiliations:** a Lecturer in Operative Dentistry, Conservative Dentistry Department, Faculty of Dentistry, Alexandria University, Egypt. Study design, performed experiments, statistical analysis, wrote the paper.; b Lecturer in Fixed Prosthodontics, Conservative Dentistry Department, Faculty of Dentistry, Alexandria University, Egypt. Performed experiments, proofread the paper.; c Assistant Professor, Dental Health Department, College of Applied Medical Sciences, King Saud University, Saudi Arabia. Proofread the paper, co-designed the experiments.; d Assistant Professor, Department of Restorative Dentistry, College of Dentistry, King Saud University, Saudi Arabia. Performed literature search, wrote the paper.; e Professor, Dental Biomaterials Department, Division Dentistry, School of Medical Sciences, The University of Manchester, Manchester, UK. Proofread the paper, co-designed the study, contributed substantially to discussion.; f Assistant Professor of Fixed Prosthodontics, Department of Prosthodontics, School of Dentistry, Taibah University, Madina, Saudi Arabia. Contributed substantially to discussion and ran statistical analysis.; g Associate Professor of Operative Dentistry, Conservative Dentistry Department, Faculty of Dentistry, Alexandria University, Alexandria, Egypt. Reviewing, editing, literature search.

**Keywords:** self-adhesive resin composites, universal adhesives, shear bond strength, microtensile bond strength, microleakage, marginal adaptation

## Abstract

**Purpose:**

This study investigated and compared the bond strengths, microleakage, microgaps, and marginal adaptation of self-adhesive resin composites (SAC) to dentin with or without universal adhesives.

**Materials and Methods:**

Dentin surfaces of 75 molars were prepared for shear and microtensile bond strength testing (SBS and µTBS). Silicon molds were used to build up direct restorations using the following materials to form 5 groups: 1. Surefil One; 2. Prime&Bond active Universal Adhesive + Surefil One; 3. Vertise Flow; 4. OptiBond Universal + Vertise Flow; 5. Scotchbond Universal + Filtek Z500 (control group). Bonded specimens were thermocycled 10,000x before being tested either for SBS or µTBS using a universal testing machine at a crosshead speed of 0.5 mm/min. Direct mesial and distal class-II cavities were created on 100 sound premolars, with the gingival margin of distal cavities placed below CEJ and restored according to the five groups. After thermocycling, microleakage scores were assessed following immersion of restored premolars in 2% methylene blue dye for 24 h, while marginal gaps and adaptation percentages were investigated on epoxy resin replicas under SEM at magnifications of 2000X and 200X, respectively. Results were statistically analyzed with parametric and non-parametric tests as applicable, with a level of significance set at α = 0.05.

**Results:**

Bond strengths, microleakage scores, microgaps, and percent marginal adaptation of Surefil One and Vertise Flow were significantly (p < 0.001) inferior to the control group. Dentin preconditioning with universal adhesives significantly increased the study parameter outcomes of Surefil One and Vertise Flow, yet they were still significantly below the performance of the control group.

**Conclusion:**

Conventional resin composite outperformed the SAC whether applied solely or in conjunction with their corresponding universal adhesives.

The growing demand for esthetic restorations has been accompanied by frequent placement of tooth-colored restorations.^39^ Direct resin composites have been used as a reliable alternative option to dental amalgam for restoring posterior teeth.^36^ Innovations in the dental industry have simplified the application of resin composites with a shift toward the bulk-fill concept. Bulk-fill composites can be applied in 4-5 mm thickness in posterior teeth, which reduces the treatment duration compared to conventional resin composites.^62^ Universal adhesives have been used in conjunction with bulk-fill resin composites to save more time and eliminate the pre-treatment of enamel and dentin surfaces. This category of dental adhesives contains specific carboxylate and/or phosphate monomers, such as 10-MDP, which bond ionically to the calcium in hydroxyapatite.^19^ Thus, they differ from current self-etch adhesives by incorporation of monomers that can bond chemically to the dental substrates.^57^

Further development in resin composite technology has led to the emergence of the self-adhesive resin composites (SAC), which do not require preconditioning of the tooth structure with dental adhesives.^8,38,44^ The composition of SAC is similar to flowable resin composites, but includes acidic (functional) monomers such as glycerol phosphate dimethacrylate (GPDM) and 10-methacryloyloxydecyl dihydrogen phosphate (10-MDP) as used in current dental adhesives.^38^ These monomers can effectively bond chemically to the tooth structure, thereby omitting the preconditioning step with a separate adhesive agent. The acidic monomers can simultaneously demineralize and infiltrate the tooth structure, resulting in micromechanical retention, while the phosphate groups of the acidic monomers can chemically bond to the hydroxyapatite of the hard dental tissues.^14^ Therefore, the use of these resin composites reduces not only the time needed to apply them compared to conventional resin composites but also the exposure to oral factors that may compromise restoration procedures.

Recently, a hybrid self-adhesive bulk-fill material that combines the cross-linking property of resin composite monomers and the self-adhesive ability of glass-ionomers has been commercialized under the brand name Surefil One (Dentsply Sirona; Bensheim, Germany). It polymerizes via a combination of chemical and light curing. The mechanical properties of SAC have been previously investigated and found to approximate the mechanical performance of conventional composites, especially in posterior teeth.^17,35,392^

Despite the time savings and ease of application of self-adhesive resin composites, scientific evidence is lacking concerning the bond strengths of this type of dental material. Further, in laboratory tests, marginal gap assessment and microleakage measurements may provide a more in-depth view of the sealing ability of these adhesive materials, which is of clinical relevance.^9^ Adaptation of a self-adhering resin composite is essential for the acidic monomers to interact with a larger area of the tooth substrate, so the higher the adaptation of SAC, the higher the hypothetical bond strength.^51^ The use of SACs in conjunction with their corresponding universal adhesives may further enhance the bond strength and marginal seal. However, this hypothesis requires testing and verification. Therefore, the current study aimed to compare the bond strengths, microleakage, and marginal gaps of SACs, with or without their corresponding universal adhesives, to a representative conventional resin composite. The null hypotheses tested were: 1. there would be no statistically significant difference between bond strengths and marginal seal of SAC and the conventional resin composites, and 2. there would be no significant difference between the bond strengths and marginal seal of SAC with or without preconditioning the tooth structure with universal adhesives.

## Materials and Methods

### Preparation of Tooth Specimens

Seventy-five intact non-restored molars and one hundred sound premolars, extracted for orthodontic or periodontal reasons, were collected from informed patients aged between 15–60 years who voluntarily donated their extracted teeth for research purposes, respecting the Declaration of Helsinki. The study was granted an ethics approval number from the Faculty of Dentistry (International No: IORG0008839). The teeth were cleaned of debris using an ultrasonic scaler, pumice, and rubber cups mounted on a low-speed handpiece. The teeth were checked under a light microscope to exclude the presence of cracks, caries, fractures, and restorations. The collected molars and premolars were stored in 0.5% chloramine-T solution at 4°C for no longer than 6 months after their extraction; the solution was regularly replenished every 4 days. When all required teeth were obtained, the storage temperature was set to 23 ± 2°C for 24 h before further testing.

The occlusal third of each molar was removed using a low-speed diamond disk (Edetal Golden S.A.W., Switzerland) under running water to expose mid-coronal dentin. Teeth presenting enamel or pulp exposure were excluded when evaluated using a stereomicroscope (ML 9300, MEIJI; Saitama, Japan) at 40X magnification. A standard smear layer was created by grinding the surface with 600-grit silicon carbide paper (waterproof silicon carbide paper, Atlas; London, UK) in one direction under running water for 30 s.

Molars and premolars were randomly assigned to 4 experimental groups and 1 control group as follows: SO: Surefil One; PB+SO: Prime&Bond active Universal Adhesive + Surefil One; VF: Vertise Flow; OB+VF: OptiBond Universal + Vertise Flow; control: Scotchbond Universal + Filtek Z500.

A detailed description of the materials used in this study with their chemical composition and instructions for use are presented in Table 1.

**Table 1:** Chemical composition and application method of the materials used in the study

Product	Manufacturer	Chemical composition	Lot No.	Application
Surefil One	Dentsply Sirona; Bensheim, Germany	Powder: silanated aluminum-phosphor-strontium-sodium-fluoro-silicate glass, dispersed silicon dioxide, ytterbium fluoride, pigments Liquid: acrylic acid, polycarboxylic acid, bifunctional acrylate, self-cure initiator, camphorquinone, stabilizer Filler loading: 77 wt%, 58 vol%	2103001383	Light cure for 20 s with an output of 1200 mW/cm^2^ Self-cure for 6 min (before further specimen processing)
Prime&Bond active universal adhesive	Dentsply Sirona	Phosphoric acid modified acrylate resin, bi- and multifunctional acrylate, acidic monomers (PENTA and MDP), isopropanol, water, initiator, stabilizer, crosslinking (N-ally), pH > 2.5	2108000044	Gentle bond agitation (20 s), air stream (5 s), light cure (10 s)
Vertise Flow	Kerr Italia; Scafati, Italy	Resin: GPDM and methacrylate co-monomers Filler: prepolymerized filler, 1 μm barium glass, nano-sized colloidal silica, nano-sized ytterbium fluoride pH=1.9	8570487	0.5-mm-thick layer created by 20 s agitation by brush, light cure (20 s), restoration build-up in increments of 2 mm or less, light cure (20 s)
OptiBond Universal	Kerr; Orange, CA, USA	GPDM self-etching adhesive monomer, mono- and bifunctional methacrylate monomers, water, acetone and alcohol, CQ-based photo-initiator system, fluoride-releasing fillers, sodium hexafluorosilicate and ytterbium fluoride	8495895	Generously apply two consecutive coats. Each coat was scrubbed in for 20 s, then dried gently with oil-free air for 10 s to evaporate the solvents, then light cure for 10 s
Filtek Z500	3M Oral Care; St Paul, MN, USA	Resin: bis-GMA, UDMA, bis-EMA, PEG-DMA, TEGMA Filler: surface-modified zirconia/silica with a median particle size of 3 μm or less; non-agglomerated/non-aggregated 20 nm surface-modified silica particles. Filler content: 82 wt%	NE34473	The thickness of the individual increments must not exceed 2.0 mm, light cure (20 s)
Scotchbond Universal	3M Oral Care	Bis-GMA, HEMA, water, ethanol, silane-treated silica, 10-MDP, 2-propenoic acid,2-methyl-, reaction products with 1,10- decanediol and P_2_O_5_, copolymer of acrylic and itaconic acid, dimethylamino benzoat (-4), CQ, (dimethylamino) ethyl methacrylate, methyl ethyl ketone, silane pH = 2.7		Gentle bond agitation (20 s), air stream (5 s), light cure (10 s)

10-MDP: 10-methacryloyloxydecyl dihydrogen phosphate; bis-EMA: ethoxylated bisphenol A glycol dimethacrylate; bis-GMA: bisphenol A glycol dimethacrylate; CQ: camphorquinone; GPDM: glycerol phosphate dimethacrylate; HEMA: 2-hydroxyethyl; H_3_PO_4_: phosphoric acid methacrylate; P2O5: phosphorous oxide; TEG-DMA: triethyleneglycol-dimethacrylate; 10-MDP: 10-methacryloyloxydecyl dihydrogen phosphate; PENTA: dipentaerythritol pentacrylate phosphate.

### Shear Bond Strength (SBS) Testing

Fifty molars with exposed dentin surfaces were selected for the SBS test in this study. Cylindrical brass molds were used to mount each molar in cold-curing acrylic resin so that the bonding surface was flush with the top surface of the acrylic resin. Embedded teeth were then assigned to 5 groups of 10 teeth each according to the different groups.

A 3-mm-high cylindrical polyethylene tube with an internal diameter of approximately 5 mm was placed on the dentin surface of each specimen and filled with each resin composite according to the manufacturers’ instructions (Table 1), and then cured for 40 s using a LED curing unit (Elipar Free Light II, 3M Oral Care; St Paul, MN, USA) with a light irradiance 1000 mW/cm^2^, which was monitored and validated by a radiometer (MARC PS, BlueLight Analytics; Halifax, NS, Canada). The tubes were removed with a sharp blade in a vertical direction and the specimens were stored in distilled water at 37°C for 24 h before being subjected to 10,000 thermocycles (5°C–55°C) with a 20-s dwell time and 10-s transfer time (SD Mechatronik; Feldkirchen-Westerham, Germany). Specimens that failed before SBS testing were recorded as pretest failures and included with an SBS of 0 MPa in calculating means for further statistical analysis.^60^

Following thermocycling, specimens were left to dry for 24 h at 37°C in an incubator before SBS testing. Bonded specimens were mounted in the jig of a universal testing machine (Tinius Olsen model no. 5ST; Redhill, Surrey, UK) and sheared with a knife-edge blade at a crosshead speed of 0.5 mm/min until they debonded. SBS was calculated in MPa by dividing the peak load at failure (in Newtons) by the specimen surface area (in mm^2^).

The mode of failure was determined using a stereomicroscope (ML 9300; MEIJI; Saitama, Japan) at 40X magnification. Failure modes were classified as: adhesive between the composite and the dentin surface; cohesive in composite; or mixed, if <80% of the surface could be classified as adhesive or mixed.^47^ Representative specimens were desiccated, sputter-coated with gold-palladium, and observed using a scanning electron microscope (JSM-IT100, JEOL; Tokyo, Japan) at a magnification of 30X.

### Microtensile Bond Strength (μTBS) Testing

Twenty-five coronally sectioned molars with exposed dentin surfaces were randomly divided into five groups of 5 (Table 1) for μTBS testing. A cubic silicon mold, with internal dimensions of 6 x 6 x 4 mm^3^, was used to build up resin composite blocks according to the studied groups (Fig 2). After building up the resin composite blocks onto the dentin surface, the teeth were stored in distilled water at 37°C for 24 h.

**Fig 1:**
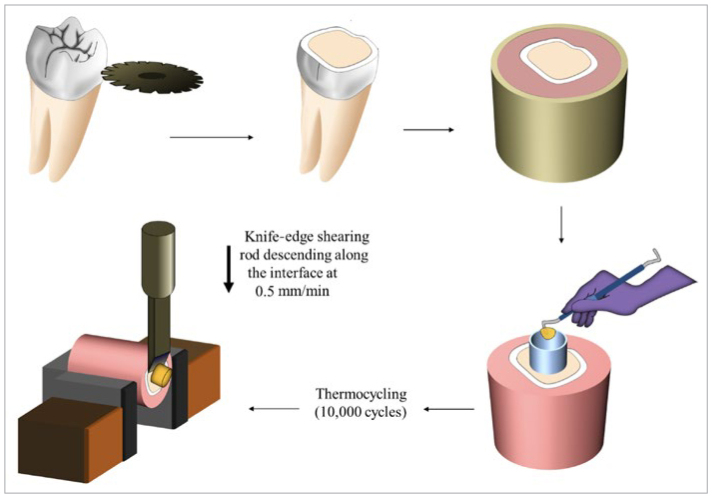
Schematic diagram showing the steps of tooth preparation for shear bond strength testing. The coronal sections of sound molars were removed to expose the coronal dentin. Teeth were then embedded in self-cure acrylic resin using cylindrical brass molds. The obtained acrylic-embedded teeth were treated according to the resin composite used with or without adhesives to obtain a cylindrical-shaped resin composite attached to the teeth. Thermocycling was then applied to the bonded specimens followed by shear bond strength testing.

**Fig 2:**
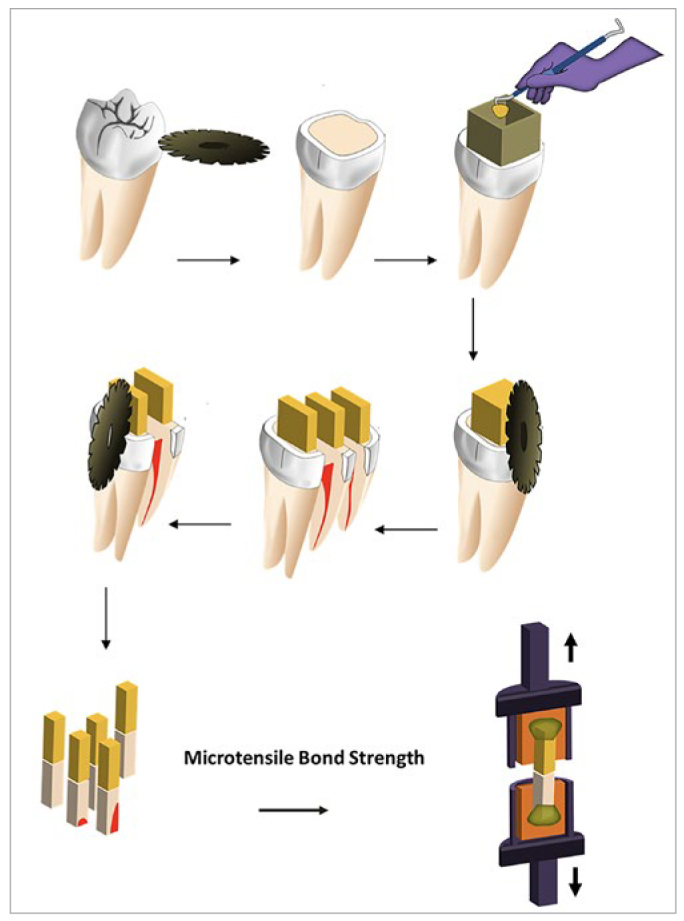
Schematic diagram of specimen preparation for microtensile bond strength testing. The occlusal third of the crown was removed exposing the mid-coronal dentin. Composite was built up using a cubic silicone mold. Composite specimens bonded to teeth were sectioned for the μTBS test. Five bars were retrieved from each tooth and mounted in the jig of the testing machine using cyanoacrylate adhesive, then tested until failure.

Each bonded sample was sectioned using a low-speed saw (Isomet 11–1180; Buehler) to produce 5 bar-shaped composite-dentin test specimens with a bonding area of approximately 1 x 1 mm^2^ for microtensile bond strength testing. Specimens were discarded if enamel was present in the section or air bubbles were seen in the composite. Five teeth from each group yielded twenty-five bars for μTBS evaluation following ISO specifications (ISO/TS 11405:2015).^4^ The bars underwent thermocycling between 5°C and 55°C with a dwell time of 20 s and a transfer time of 10 s. After thermocycling, the bars were glued to the microtensile grip of a universal testing machine (MTS 858 Mini Bionix II with control and acquisition software: MTS Systems; Eden Prairie, MN, USA) using cyanoacrylate glue (Loctite 406; Dublin, Ireland). Force was applied in tension at 0.5 mm/min and a 90-degree angle until failure occurred. The fracture load (N) was recorded and the μTBS (in MPa) was calculated according to the formula:^32^

μTBS = L/A

where L is the load (in N) required to fracture the specimen, and A is the interfacial area (mm^2^).

Modes of failure of all specimens were detemined using the stereomicroscope (ML 9300) at 40X magnification. Two representative fracture surfaces were processed for examination in the SEM (JSM-IT100). The failure mode was classified as one of five types following the microscopic evaluation of the fractured surfaces: A: adhesive failure (fracture in the adhesive layer); C: cohesive failure of the resin composite or dentin; M: mixed failure where the fracture site involved more than one substrate; P: pre-test failure (fracture occurred before the specimen was tested); G: failure outside the test region where the specimen was glued to the attachment device.^7^

### Microleakage and Marginal Gaps

One hundred premolars were used, distributed randomly into the 5 groups (n=20). Coarse diamond stones (846KR, Komet Italia; Milano, Italy) were used to prepare two separate class-II cavities in each tooth with a 90-degree cavosurface angle and straight walls with rounded internal line angles and no retentive grooves. Cavities were finished with fine-grained burs of the same shape (8846KR, Komet Italia). Cutting and finishing burs were replaced every 5 cavity preparations. Mesial and distal cavities were created with dimensions of 4 mm width (buccolingually) and 2 mm depth (mesiodistally), which were checked and verified using a WHO periodontal probe (Hu Friedy; Chicago, IL, USA) and a 5X-magnification loupe under LED illumination. The gingival step was placed 1 mm above (on the enamel margin) and below (on a cementum/dentin margin) the cementoenamel junction for the mesial and distal cavities, respectively (Fig 2).

A circumferential transparent matrix (Automatrix MT, Dentsply Sirona) was adjusted around each cavity for adequate conformation of the restoration walls. A water-resistant marker was used to mark the appropriate thickness of future resin composites depending on the study group (as described in Table 1). The marks were placed at 2 mm distances from the gingival step to the occlusal margin. A digital caliper was used to guide the placement of marks on the outer surface of the matrix. Resin composites were built up incrementally, so that each increment reached the mark on the transparent matrix, then light cured for 40 s using an LED curing unit (Elipar S10, 3M Oral Care) with 1100 mW/cm^2^ irradiance. The light tip was placed in contact with the top edge of the transparent matrix. Matrices were removed and the margins of restored teeth were finished with coarse, medium, fine, and superfine disks (Sof-lex, 3M Oral Care). Teeth were immersed in distilled water for 21 days at 37°C^17^ and then subjected to thermal aging via 10,000 thermocycles (5°C–55°C) with a 20-s dwell time and a 10-s transfer time (Mechatronik).

Half of the thermocycled teeth (n=10/group) were left to dry and then covered with two coats of nail varnish except for the restoration site and the surrounding 1-mm margin. The dental apices were sealed with cyanoacrylate glue (Loctite) to prevent penetration of the dye through the apex. Specimens were then immersed in a 2% methylene blue dye at 37°C for 24 h following ISO standards (ISO/TS11405). After 24 h, the specimens were washed with abundant water for 5 min. The roots were shortened up to 3 mm below the restoration margins, while the crowns were sectioned lengthwise (mesiodistally) with a double-sided 0.2-mm diamond disk (Struers; Ballerup, Denmark) mounted in a low-speed handpiece and with abundant water irrigation. The sectioned surfaces were polished with silicon carbide papers under a stream of water for 2 min and later dried for stereomicroscope observation (ML 9300, MEIJI) at 20X magnification to register the degree of marginal microleakage. Both the statistician and the operator who performed the readings under the stereomicroscope were blind to the group assignment. The scoring system, provided by the International Organization for Standardization PD (ISO/TS 1145:2015),^4^ was employed to measure the extent of the methylene blue dye penetration (Table 2).

**Table 2:** Scoring system to quantify marginal microleakage

Score	Microleakage
0	No dye penetration
1	Penetration into the enamel of cavity wall
2	Penetration into the dentin of cavity wall without including the pulpal wall of the cavity
3	Penetration including the pulpal wall of the cavity

The other half of the thermocycled teeth (n=10/group) were cut in an oro-vestibular direction between the mesial and distal cavities using a precision saw (Isomet 11–1180, Buehler; Lake Bluff, IL, USA). The resulting specimens were mounted on self-cured acrylic-resin–filled plastic cylindrical molds so that the flat surfaces of the sectioned teeth protruded from the acrylic resin. Specimens were then ultrasonically cleaned and air dried. Replicas of the specimens were obtained via addition silicon impressions, then poured with epoxy resin (EpoFix, Struers; Ballerup, Denmark). The obtained replicas were sputter-coated with Au/Pd for examination by SEM (magnification: 2000X) of marginal gaps at the gingival margin. Five specimens from each group were examined. The whole gingival margin/restoration interface was assessed separately in the mesial and distal restorations and the marginal gaps were scored as follows:^2^ 0: no marginal gap; 1: maximum marginal gap < 30 μm; 2: maximum marginal gap > 30 μm.

Further, the whole length of the restoration-tooth interface was measured using the SEM tracing tools at a magnification of 200X, and the lengths of continuous margin between the restoration and the tooth were recorded. The marginal adaptation percentage of each restoration was calculated using the following equation:^3^


$$\text{Percentage of marginal adaptation}(\%)=\frac{\text{Sum of length of continuous restoration along the interface with the tooth}}{\text{The total length of restoration}-\text{tooth interface}}\times 100$$

### Statistical Analysis

Statistical analysis was performed using the statistical software package SPSS version 22 (Chicago, IL, USA). Quantitative data were checked for normality and homogeneity of variance using the Kolmogrov-Smirnov and Levene tests, respectively. Shear and microtensile bond strengths and the percentages of marginal adaptation were found to be normally distributed (p = 0.92, 0.31 and 1.2, respectively), so that means and standard deviations (SD) were calculated, and parametric tests were adopted. One-way ANOVA and Tukey’s post-hoc test were used to compare the statistical significance (α = 0.05) of the mean bond strengths and the mean percentages of marginal adaptation between the groups, while the mean percentages of marginal adaptation between mesial and distal restorations for the same group were statistically compared using the independent t-test (α = 0.05). The normality of the microleakage scores was violated; hence, non-parametric tests were used. Microleakage and marginal gap scores between different groups for the proximal boxes mesially or distally were statistically compared using the Kruskal-Wallis test and Mann-Whitney pairwise comparison (α = 05). The microleakage scores between the mesial and distal boxes for the same group were compared analysed using the Wilcoxon test with a level of significance set at α = 0.05.

## Results

### Bond Strengths

Descriptive statistics for SBS and µTBS bond strengths, as well as the frequency of failure modes are given in Table 3. One-way ANOVA showed a statistically significant difference between the mean SBS between groups (p < 0.001). The highest mean SBS (23.7 ± 1.4, p < 0.001) existed in the control group (Scotchbond Universal + Filtek Z500), followed by OB+VF and PB+SO groups (12.0 ± 1.3 MPa and 9.3 ± 1.1 MPa, respectively). Universal adhesives significantly increased the SBS of SAC (p < 0.001). A similar trend among the studied groups was also found when comparing the mean µTBS (p < 0.001) of the five groups. Surefil One showed predominantly adhesive failures after SBS and µTBS testing. The control group showed predominantly mixed failures in the SBS test, and most of the specimens failed cohesively in the tooth structure outside the test region during µTBS testing (Table 3).

**Table 3:** Descriptive statistics (mean ± SD) of SBS and µTBS with their failure modes

Restoration	SBS (MPa) Mean ± SD	Failure mode			SBS (MPa) Mean ± SD	Failure mode				
		A	C	M		A	C	M	P	G
Surefil One	3.56 ± 0.65A	10	0	0	10.9± 5.2A	18	0	0	7	0
Prime&Bond active universal adhesive + Surefil One	9.33 ± 1.08B	4	1	5	22.5 ± 6.5BC	12	3	7	3	0
Vertise Flow	3.39 ± 0.91A	8	0	2	13.1 ±5.9AB	15	1	4	2	0
OptiBond Universal + Vertise Flow	11.95 ±1.29C	3	3	4	26.1 ± 6.8C	9	4	6	0	2
Scotchbond Universal + Filtek Z500	23.69 ±1.37D	0	1	9	58.4 ±7.3D	0	2	8	0	15

Similar superscript capital letter indicates no statistically significant difference (p > 0.05) between groups in columns. A: adhesive failure; C: cohesive failure; M: mixed failure; P: pre-test failure; G: failure outside the test region.

### Microleakage

The distribution of microleakage scores for each group is presented in Fig 4. Representative images for the different study groups are shown in Fig 5. For the mesial box, the Kruskal-Wallis test revealed a significant difference in the microleakage scores between groups (p < 0.001). Mann-Whitney pairwise comparisons showed a significant difference in the microleakage score between SO and both control (p ≤ 0.001) as well as OB+VF groups (p < 0.001). A significant difference was also found between PB+SO and control (p = 0.003) as well as OB+VF (p = 0.03).

**Fig 3:**
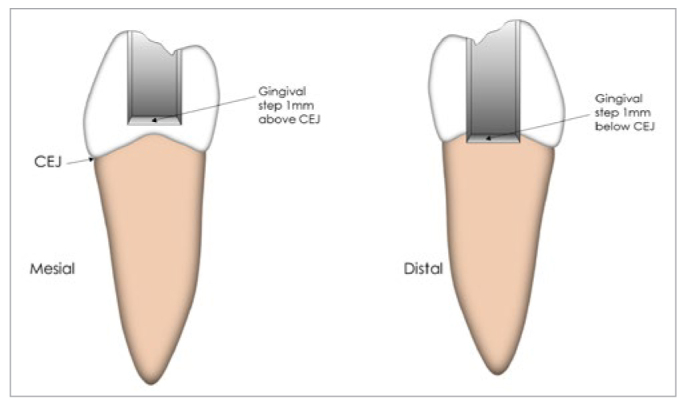
Schematic diagram showing the class-II cavity designs on the mesial and distal surfaces of a premolar. The gingival step was located 1.5 mm above the CEJ on the mesial surface and 1.5 mm below the CEJ on the distal surface.

**Fig 4:**
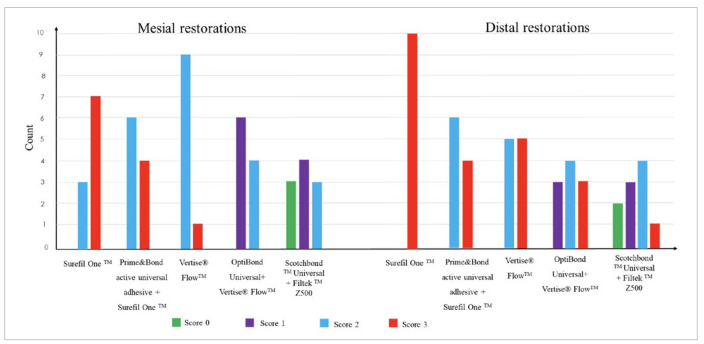
Microleakage scores of mesial and distal class-II restorations for each group.

**Fig 5:**
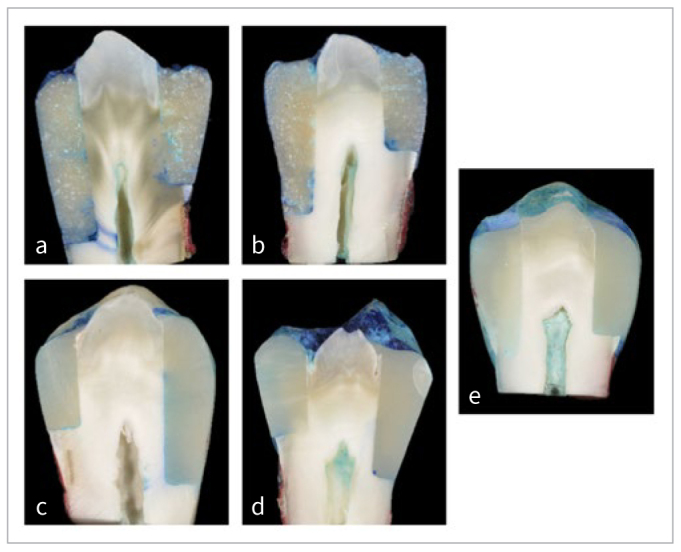
Microleakage of resin composite restorations in mesial and distal class-II cavities; a) Surefil One (score 2 mesially and 3 distally); b) Prime&Bond active universal adhesive + Surefil One (score 2 mesially and distally); c) Vertise Flow (score 1 mesially and score 2 distally); d) OptiBond Universal+ Vertise Flow (score 2 mesially and score 3 distally); e) Scotchbond Universal + Filtek Z500 (score 0 mesially and distally).

In distal boxes for all studied groups, statistical significance was found between the control group and SO, PB+SO, VF, and OB+VF groups (p = 0.000, 0.044, 0.013, and 0.032, respectively).

The Wilcoxon non-parametric test showed no significant difference between microleakage scores of mesial and distal restorations for the same material group, except for the VF (p = 0.04) and OB+VF (p = 0.008) groups.

### Marginal Gaps

The marginal gap scores are presented in Table 4. The Kruskal-Wallis test showed a statistically significant difference between the groups (p < 0.001) for both mesial and distal restorations.

**Table 4:** Marginal gap scores and percentages of marginal adaptation among the 5 groups

Restoration	Mesial Scores			Percentage of marginal adaptation Mean ±SD	Distal Scores			Percentage of marginal adaptation Mean ±SD
	0	1	2		0	1	2	
Surefil One	0	10	0	57.9 ± 6.5^Aa^	0	10	0	51.3 ±8.1^Aa^
Prime&Bond active universal adhesive + Surefil One	1	9	0	69.1± 5.2^Ba^	0	10	0	64.7 ± 9.2^Ba^
Vertise Flow	2	8	0	89.1 ± 4.4^Ca^	1	9	0	82.1± 3.3^Ca^
OptiBond Universal+ Vertise Flow	7	3	0	93.2 ± 3.0^Ca^	3	7	0	90.0 ± 5.0^CDa^
Scotchbond Universal + Filtek Z500	9	1	0	98.1 ± 2.1^Da^	7	3	0	97.1 ± 3.6^Da^

Same superscript capital letter indicates no significant difference (p > 0.05) between the mean percentage of marginal adaptation in columns; same superscript lower-case letter indicates no significant difference (p > 0.05) between the mean percentage of marginal adaptation of mesial and distal restorations for the same group.

In mesial restorations, Mann-Whitney pairwise comparisons showed no significant difference between the marginal gap scores of the groups except between OB+VF (p = 0.000) and control (p = 0.000) and the other groups. A similar trend was found in the distal box, except for a significant difference between the control group and all other groups. The Wilcoxon non-parametric test showed no significant difference between microleakage scores of mesial and distal restorations (p > 0.05).

**Fig 6:**
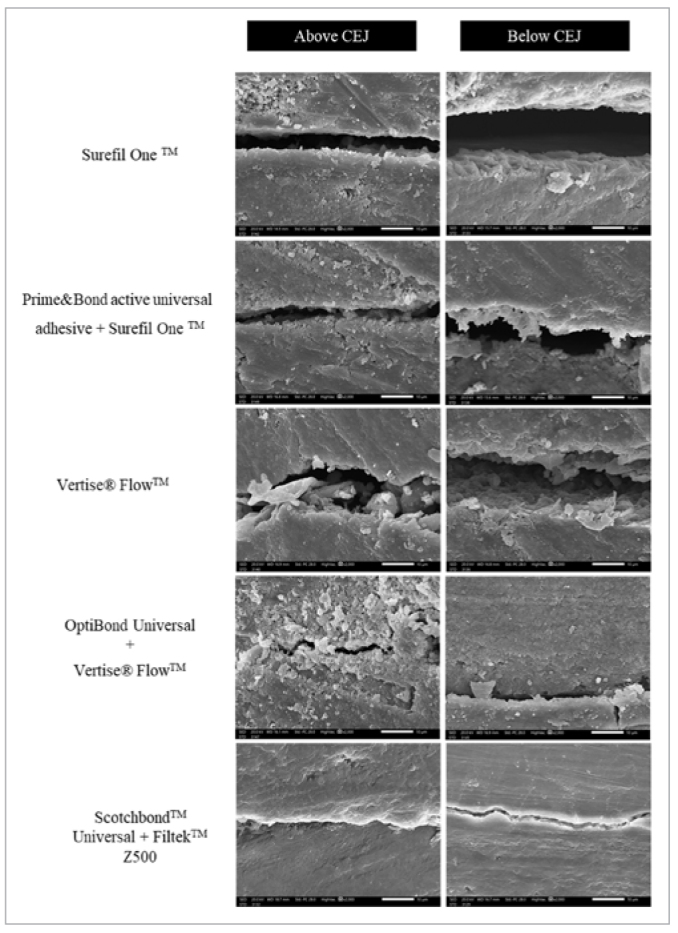
SEM images showing the marginal gaps at the gingival steps of mesial and distal class-II restorations for various groups (2000X magnification).

All investigated teeth showed either a score 0 or 1 with no occurrences of score 2, irrespective of the restoration type and location. Generally, there were larger marginal gaps for all groups below than above the CEJ, indicating better marginal adaptation to enamel than to cementum (Fig 6). The highest marginal gaps were found in the SO group below the CEJ (14.7–20.6 µm), followed by VF below the CEJ (12.2–14.6 µm), which decreased when the restorations were placed after application of their corresponding universal adhesives in PB+SO and OB+VF groups (4.8–6.2 µm and 1.6–3.0 µm, respectively). The lowest values were found in the control group, ranging from 1.0–1.1 µm and 2.1–2.9 µm above and below CEJ, respectively.

One-way ANOVA showed statistically significant differences between the percent marginal adaptation among restorative material groups (p < 0.001). The control group had significantly better cavity adaptation than did the other restorative material groups (p < 0.001) in both mesial and distal boxes, and similarly between SO and PB+SO (p = 0.010). A significant difference was found between VF and OB+VF (p = 0.023) only in mesial restorations. Independent t-tests showed a non-significant difference in the marginal adaptation of different material groups between mesial and distal restorations (p > 0.05).

## Discussion

Manufacturers of dental materials have attempted to simplify the placement of the technique-sensitive resin composites by creating universal adhesives, SACs, and bulk-fill SACs. This study investigated and compared the bond strengths, microleakage, marginal gaps, and marginal adaptation of SACs, with and without their corresponding universal adhesives, to a conventional resin composite. A statistically significant difference between groups was found, and hence the null hypotheses were rejected.

In this study, an array of different SACs with different bonding strategies was evaluated to indicate how these materials – with different adhesive approaches – may behave in clinical situations. Five clinically crucial outcomes were evaluated, i.e., SBS, µTBS, microleakage, marginal gaps, and adaptation. Although clinical trials remain the conclusive approach to determine the performance of dental materials, preclinical assessment through laboratory testing is still an essential prerequisite for the evaluation of adhesive dental materials.^61,65^ Numerous data can be collected and investigations performed under standardised conditions with the absence of the confounding factors that are present in clinical studies.^48^

The rationale for conducting shear bond strength testing was based on the premise that the higher the SBS of restorative materials to the tooth structure, the higher the expected resistance to functional occlusal forces in the clinical situation.^15^ However, there may be some limitations inherent in SBS testing, e.g., the non-uniform stresses generated by the shearing rod over the bonded specimens and the frequent cohesive failure of dentin substrates rather than at the adhesive interface.^54^ Despite being more technique-sensitive and time-consuming, microtensile bond strength testing is reliable tool with high discriminative power to assess the bond strength between dissimilar materials.^13^

### Bonding Effectiveness of SACs

Self-etch adhesive agents bond to tooth substrates through a chemical bond between the Ca^+2^ ions of the tooth structure and the adhesive monomers of the bonding agent.^52^ Resin composites with self-adhesive characteristics bond to tooth structure in a similar way.^11^ Self-etch adhesives have been shown to increase the bond strength of resin composites to tooth structure in previous studies.^43,50^ However, the results of the current study showed significantly compromised bond strengths to tooth structure compared to a conventional resin composite. A possible explanation could be the higher viscosity and poor wettability of SACs compared to the self-etch adhesives.^19,40^ High viscosity and poor wettability might have influenced the total area of tooth structure that was covered by the resin composites, and hence reduced interaction between the acidic monomers and Ca^+2^.^40^ Further, SACs contain a smaller amount of acidic monomers than do self-etch adhesives, which could explain the significantly lower bond strengths of the SACs used in this study.^22^

Ultimately, bonding between an adhesive-containing material and tooth structure depends to a great extent on the ability of the adhesive to remove the smear layer, spread widely, and infiltrate into the exposed dentinal tubules.^22^ The highly viscous nature of the SACs might have impeded the proper penetration of the material into dentinal tubules, which were possibly blocked by incomplete removal of smear layer. Despite a chemical composition similar to that of self-etch adhesives, SACs could have instead interacted superficially with the tooth substrates, leading to compromised bond strengths.

Self-adhesive resin composites, in conjunction with universal adhesives, significantly increased dentin bond strength and the percentage of marginal adaptation, and reduced microleakage scores. A previous study reported a significant increase in bond strengths of SACs to dentin when the latter was preconditioned with universal adhesives.^16^ The improvement in bond strengths could be attributed to the higher wettability of universal adhesives, which allows better micromechanical retention and wider chemical interaction between the acidic functional monomer in the universal adhesive and calcium in dentin. Further, the high resin-monomer content in SACs facilitates strong co-curing with the universal adhesive.^10,30^ In addition, the universal adhesive acted as an intermediary stress reliever to partially compensate shrinkage stress. While universal adhesives generally have a rather thin film thickness, their separate application and polymerization may have contributed to better withstanding high polymerization shrinkage. Such a stress-absorbing role of adhesive cannot be assumed by self-adhesive materials.^5^

Vertise Flow was used in the current study as a representative example of SACs. It chemically bonds to tooth surfaces by two mechanisms: the primary bonding mechanism is based on a chemical interaction between the calcium ions in the tooth and the functional phosphate groups in the GPDM monomers found in the resin, whereas the secondary bonding mechanism consists of micromechanically etching the tooth facilitated by the low pH of the resin material, which is similar to that of numerous self-etching materials.^66^ Although VF incorporates adhesive technology found in Optibond products to create bonds to the tooth structure, adding other fillers may reduce the bond strength of VF, as discussed by Bektas et al.^46^ Previous studies reported low bond strengths of Vertise Flow to enamel and dentin. The acidity of Vertise Flow (pH = 1.9)^56^ is below that of phosphoric acid etchant; hence, an expectedly weaker demineralization effect on tooth substrates and fewer microporosities may have decreased the bond strengths of the material, regardless of the tooth substrate, which is in agreement with previous findings.^23,26,28^ Further, thermocycling might have weakened the interface between Vertise Flow and dentin, possibly due to hygroscopic expansion and solubility phenomena of Vertise Flow in the presence of water, as described in previous studies.^23,66,67^

Despite the presence of the hydrolytically stable Modified Polyacid System (MOPAS) monomer in Surefil One that would be expected to promote adhesion and act as a copolymerizing crosslinker in the cured material,^33^ Surefil One had weak self-adhesion to dentin (3.56 MPa), which did not significantly differ from VF on untreated dentin (3.39 MPa). The pH of Surefil One is 2.1 directly after mixing and 3.2 after 6 min. The material then gradually becomes pH neutral. Surefil One could have superficially demineralized dentin with expected inferior bonding performance; of note, Surefil One specimens showed the highest frequency of pre-test failures. This could be due to the vibration of the cutting saw during the sectioning of the teeth, and possible voids near the adhesive interface. These factors might have weakened the already weak adhesive layer, promoting the failure of specimens before testing.^7^ These findings agree with those of previous studies reporting low bond strengths of SACs (3.4–17.7 MPa) and a high rate of pretest failures (10%–66.7%).^12,19,50^ However, François et al^16^ reported higher SBS of Surefil One to dentin (14.0 ± 3.4 MPa), which increased significantly when dentin was pre-conditioned with universal adhesive (20.9 ± 4.1 MPa). However, the authors did not subject the bonded specimens to thermocycling before SBS testing, which might explain the contradiction with the findings of the present study. Thermal aging of 10,000 cycles can deteriorate the resin-dentin interface, leading to a significant decrease in bond strengths, as supported by previous works.^23,37,68^ SBS of less than 10 MPa is significantly lower that those previously reported for composites with universal adhesives in the self-etch mode under the same bonding test conditions as specified in ISO 29022.^59^

The control group obtained the highest bond strengths compared to other groups. Scotchbond Universal has higher wettability and lower viscosity compared to SACs, due to its content of hydrophilic solvents such as ethanol and water. Ethanol promotes displacement of water from the exposed collagen fibers in dentin and infiltration of resin monomers in the interfibrillar dentin.^42^ With the additional role of functional and acidic monomers (10-MDP) within the universal adhesive, better interaction with the dentin was expected and thus higher bond strengths.^57^

It is well-known that failure mode is a reflection of bonding effectiveness, and that adhesive failure reflects low bond strengths.^ 49^ This study reported predominantly adhesive failure of SACs when applied solely to dentin, consistent with the reported low bond strengths and in agreement with previous findings.^56^ Mixed failure was frequent in the Scotchbond Universal/Filtek Z500 group, indicating that the mechanical resistance and structural strength of dentin and bonded Filtek Z500 were the weakest link in the system. The bond strength between the two substrates might have already exceeded the intrinsic strength of either of dentin or the bonded Filtek Z500 resin composites, leading to either cohesive or mixed adhesive/cohesive failures.^28^ Scotchbond Universal contains 10-MDP functional monomer, which ionically bonds to hydroxyapatite of dentin substrates and improves the bond strength at the adhesive joint, in agreement with Souza et al.^57^ Adhesive failures, however, occur when the fracture toughness of the bonded substrates exceeds the bond strength of the adhesive joint at the interface.^27^ In the current study, debonded specimens of Vertise Flow or Surefil One showed predominantly adhesive failure after SBS testing. Their significantly lower SBS (3.39 ± 0.91, 3.56 ± 0.65, respectively) might reflect the weak bonding interface rather than the fracture toughness of the bonded substrates. Similar materials showed higher SBS when used in conjunction with their universal adhesives, with less frequent adhesive failure, indicating improved bond strength at the adhesive joint, which agrees with a previous study.^58^

### Marginal Quality of SAC

A tight marginal seal is a primary objective for direct restorative materials. Insufficient bonding to tooth substrates will create microgaps due to polymerisation shrinkage of resin composites, which facilitates bacterial seepage, consequent recurrent caries, and eventually a dislodged restoration.^55^ Therefore, it was essential to investigate the microleakage of SAC resin composites in class-II cavities with deep or shallow gingival margins to predict how the material would perform in the clinical situation.

Microleakage was more severe (score 3) at the cervical margins located in dentin than at the cervical margins located in enamel (score 1). The microleakage results are consistent and expected based on the lower percentage of continuous margins for dentin. Similarly, previous studies showed that microleakage tends to be higher in dentin than in enamel.^1,21,41^ Dentin is biologically more variable than enamel, which makes it a more difficult substrate upon which to obtain high bond strength with the adhesive, which must resist thermal stresses and the interfacial stresses generated by polymerization shrinkage of the composite resin. Our results support the research of Kalmowicz et al,^ 29^ which found that the microleakage in enamel was significantly lower compared to that in dentin, regardless of the material, C-factor, or insertion technique. Furthermore, the distance from the light-curing source on the occlusal side to the gingival margin is greater in distal boxes than mesially, with an expected higher loss of light energy in the deepest layers of distal restorations, which corroborates the results of previous studies.^6,20^

Despite the ongoing research and development of dental adhesives by the manufacturers, a 100% tight seal between the restoration and tooth substrate can still not be clinically achieved. Preconditioning of tooth structure separately by acid etching or the application of primers and bonding agents have repeatedly shown higher bond strengths with resin composites, and longer service in clinical practice compared to the simplified all-in-one adhesives.^64,65^ Recent developments in universal adhesives, however, have disproven the theory that all-in-one adhesives invariably result in poor adhesion of direct resin composites to tooth substrates.^17,18^

The current study reported poor bond strengths, marginal adaptation, and larger microgaps of the recent bulk-fil Surefil One compared to the conventional resin composite. In a previous study, Surefil One has demonstrated good marginal quality, high wear, and fracture resistance after thermomechanical fatigue tests comparable to that of dental amalgam.^17^ However, the good mechanical performance of Surefil One should not be considered to outweigh its poor bond strengths to tooth substrates. Unlike dental amalgam, resin composites rely on robust adhesion to tooth structure for long-term, durable service in the patient’s mouth.^65^ The rationale for using SACs in dental practice has been to provide the clinician with a less technique-sensitive material, without sacrficing other properties necessary for successful restorations.

Surefil One has been previously investigated for its sealing ability in class-II cavities. After 500,000 cycles of thermomechanical aging, Surefil One showed gap-free margin percentages of 48±15% and 53±17% with dentin and enamel, respectively, as reported by Frankenberger et al.^17^ This finding agrees with the findings of the current study, which showed similar marginal adaptation percentages of 57.9±6.5 and 51.3±8.12) after 10,000 thermocycles for mesial and distal cavities, respectively.

Scotchbond Universal, used in the control groups, showed the best marginal quality compared to other groups. Previous studies recommended the use of this 10-MDP-based universal adhesive in class-II cavities and reported reduced marginal leakage at the resin/dentin interface.^31,45^ The functional primer (10-MDP) can ionically bond to the abundant hydroxyapatite around the collagen fibrils and form stable and water-insoluble MDP-Ca–salt nanolayers.^63^

The occlusal surface of the restoration is the region closest to the light-curing unit and thus has the smallest loss of energy density during curing.^6^ However, class-II restorations are challenging, particularly when the cervical margins are below the CEJ. Margins below the CEJ make it difficult to achieve good results for marginal sealing, polishing, and longevity.^34^ Thus, the present study also evaluated the microleakage at the cervical margins, including the mesial margin of dentin and the distal margin of enamel. Moreover, composite marginal integrity might be affected by several factors, including the cavity size and geometry, the composite resin’s physico-mechanical properties, the layering protocol, and the polymerization technique.^25,26^ In the present study, we standardized the cavity size, the adhesive technique, and the polymerisation technique, so that only the resin variable would influence the results and whether the respective universal adhesive was used or not.

The limitations of the current study involve the lack of in-depth assessment of the hybrid layer using SEM for better evaluation of the morphological interaction between SAC and dentin at the adhesive joint. The adhesives used in the study were limited to the all-in-one category. Other two-step adhesives, and different conditioning methods of dentin might lead to different interactions between SAC and tooth substrates. Further, physical and mechanical properties of the self-adhesive resin composites need further investigation to provide better understanding of the polymerization of these materials. The necessity of preclinical screening to test restorative materials does not replace the more realistic outcomes of clinical trials. Randomized controlled clinical trials with long-term follow-up may be more appropriate to monitor the clinical performance of SAC. Apart from a previous one-year follow-up clinical study,^53^ which reported clinically promising results of Surefil One, the evidence is still insufficient for deciding to use SAC confidently in dental practice.

## Conclusion

Conventional resin composites, bonded to tooth substrates by their corresponding universal adhesives, outperformed the SACs in all the studied parameters, i.e, SBS, µTBS, microleakage, microgaps, and marginal adaptation. Although the use of universal adhesives significantly improved bond strengths, microleakage, microgaps, and marginal adaptation of SAC, their performance is still considerably below that of conventional composites.

## Clinical relevance:

In terms of bonding procedure, bond strength, and marginal adaptation quality, conventional resin composites are more technique sensitive, yet with higher performance compared to the new self-adhesive bulk-fill resin composites in the all tested in-vitro parameters.
